# A human skeletal muscle interactome centered on proteins involved in muscular dystrophies: LGMD interactome

**DOI:** 10.1186/2044-5040-3-3

**Published:** 2013-02-15

**Authors:** Gaëlle Blandin, Sylvie Marchand, Karine Charton, Nathalie Danièle, Evelyne Gicquel, Jean-Baptiste Boucheteil, Azéddine Bentaib, Laetitia Barrault, Daniel Stockholm, Marc Bartoli, Isabelle Richard

**Affiliations:** 1 Généthon CNRS UMR8587, 1, rue de l'Internationale, Evry 91000, France; 2 Present address: Aix-Marseille Univ and Inserm, UMR 910, Faculté de Médecine Timone, Marseille 13385, France

**Keywords:** Interactome, Muscular dystrophies, Yeast-two hybrid

## Abstract

**Background:**

The complexity of the skeletal muscle and the identification of numerous human disease-causing mutations in its constitutive proteins make it an interesting tissue for proteomic studies aimed at understanding functional relationships of interacting proteins in both health and diseases.

**Method:**

We undertook a large-scale study using two-hybrid screens and a human skeletal-muscle cDNA library to establish a proteome-scale map of protein-protein interactions centered on proteins involved in limb-girdle muscular dystrophies (LGMD). LGMD is a group of more than 20 different neuromuscular disorders that principally affect the proximal pelvic and shoulder girdle muscles.

**Results and conclusion:**

The interaction network we unraveled incorporates 1018 proteins connected by 1492 direct binary interactions and includes 1420 novel protein-protein interactions. Computational, experimental and literature-based analyses were performed to assess the overall quality of this network. Interestingly, LGMD proteins were shown to be highly interconnected, in particular indirectly through sarcomeric proteins. In-depth mining of the LGMD-centered interactome identified new candidate genes for orphan LGMDs and other neuromuscular disorders. The data also suggest the existence of functional links between LGMD2B/dysferlin and gene regulation, between LGMD2C/γ-sarcoglycan and energy control and between LGMD2G/telethonin and maintenance of genome integrity. This dataset represents a valuable resource for future functional investigations.

## Background

The skeletal muscle tissue with its movement generation capacity is the organ of voluntary action but it plays also a major role in metabolic homeostasis. It is composed of long multinucleated cells, the myofibers, which possess a highly structured organization to ensure the dynamics and coordination of muscle contraction and resistance to resulting physical stresses. Notwithstanding its very well organized structure, the muscular tissue presents an important plasticity, which is necessary for adaptation to physical and metabolic constraints. These combined characteristics of structure and plasticity depend on the concerted action of protein complexes and of metabolic and signaling pathways. These features together with the complexity of the skeletal muscle organization and its connection with human disorders makes it an interesting tissue for proteomic studies aimed at understanding functional relationships of interacting proteins in both health and diseases.

During the last decade, an increasing number of studies have been carried out to produce and analyze large-scale protein-protein interaction networks in various bacterial and eukaryotic model systems. Several studies have investigated the human interacting-proteins network, either at a genome-wide scale [1-3] or with the aim of exploring a targeted interaction network [4-6]. Remarkably, several disease-targeted projects have proven to offer a very powerful strategy to address the role and function of the proteins involved, as exemplified by Huntington’s disease [5] and in spinocerebellar ataxia [6]. These works have capitalized on several high-throughput technologies (yeast-two hybrid (Y2H) system and Tandem Affinity Purification) to detect binary protein-protein interactions (PPI) or protein complexes, and have combined them with computational methods to propose “interactome” networks.

As an approach towards the identification of skeletal muscle functional networks, we undertook a large-scale study to establish a proteome-scale map of protein-protein interactions centered on proteins involved in limb-girdle muscular dystrophies (LGMD). LGMD is a group of neuromuscular disorders with autosomal dominant (LGMD1) or recessive (LGMD2) inheritance modes that principally affect the proximal pelvic and shoulder girdle muscles (for a recent review see [7]). More than twenty different genetic entities were identified so far but it is estimated that 30-40% of patients clinically diagnosed with LGMD still do not have a genetic signature of their disease [8] with at least 25% of families who are not linked to any known locus and 40% of isolated cases with no detected mutation in any known LGMD gene. The known LGMD-causing genes encode proteins expressed in a variety of cellular compartments and involved in diverse biological functions that are not yet fully understood. Yet, the present knowledge has highlighted the importance of the components of the dystrophin-glycoprotein complex and α-dystrophycan glycosyltransferases for membrane stability, and the implication of other LGMD proteins in processes such as regulation of sarcomere structure or nuclear stability, for the survival of the muscle fibers [9]. No curative treatment is currently available for LGMD, which pleads for the elucidation of the pathological mechanisms implicated in the diseases in order to propose innovative therapeutic approaches.

In this context, we selected a large-scale strategy to identify novel protein-protein interactions that could shed light on the biological processes at the origin of LGMD pathogenesis. We selected 13 LGMD-causing proteins and related proteins and ran high-throughput Y2H assays to build a first interactome network that we further expanded by performing additional secondary and tertiary Y2H screenings with new bait proteins of interest identified as preys in the primary screenings. The resulting LGMD-centered interaction network was established by combining results of 87 screenings based on 76 different bait proteins and incorporates 1018 proteins connected by 1492 direct binary interactions. Using both experimental and bioinformatics tools, we assessed the overall quality of this Y2H network and isolated a high-confidence (HC) sub-network of 705 PPIs associating 497 proteins. The Y2H LGMD-centred network and its HC sub-network were compared to a literature-based network. Gene Ontology (GO) term analysis showed that the LGMD-centered interactome and especially its HC sub-network, is much more specifically enriched in proteins associated with the muscular tissue and the cytoskeleton than the literature-based dataset. Among the interesting outcomes of our study are the strong inter-connectivity of the LGMD proteins which, in addition to several direct links, present a number of indirect associations thanks to sarcomeric proteins, the identification of candidate genes for orphan neuromuscular disorders (NMDs) and the discovery of new possible functions for LGMD proteins. In particular, we suggest the existence of functional links between LGMD2B/dysferlin and gene regulation, between LGMD2C/γ-sarcoglycan and energy control and between LGMD2G/telethonin and maintenance of genome integrity.

The protein interactions from this publication have been submitted to the IMEx ( http://www.imexconsortium.org/) consortium through IntAct (pmid 22121220) and assigned the identifier IM-16425. The entire network can be browsed using the PIMRider software at http://pimr.hybrigenics.com.

## Methods

### Y2H bait design

Bait design was organized in three successive rounds in which primary, secondary and tertiary baits were selected. Structural and functional domain predictions from TM-HMM [10], SignalP3.0 [11] or PFAM (PFAM 23.0, release 19/08/2008 [12]) were used to exclude hydrophobic trans-membrane domains, signal peptides and transcriptional trans-activation domains from bait constructs. In addition, to favor the identification of novel protein-protein interactions, we usually selected regions on the proteins that had not been previously documented for their functional role. For selection of secondary and tertiary bait proteins and for design of their bait sequences, we used additional bibliographic searches and other criteria computed from our Y2H results such as the Predicted Biological Score (PBS) categories and information from Selected Interacting Domains (SID; see below section “Identification of interacting fragments and scoring of the interactions”). Some examples of bait candidates that came to our attention included targets of choice for therapeutic strategies such as proteins that participate in signaling pathways, proteins involved in various forms of myopathies or proteins expressed in typical muscle cellular compartments such as the sarcomere or sarcoplasmic reticulum.

### Bait cloning and library construction

Bait sequences were PCR-amplified from MRC Gene Service or Invitrogen plasmids or from a random primed cDNA library obtained by reverse transcription of a poly(A) RNA library isolated from adult (Ambion AM7983) or 18-19 week-old fetal (Stratagene #778020) human skeletal muscles. Bait PCR products were cloned in the pB27 plasmid, a plasmid derived from the original pBTM116 [13], as a LexA C-terminal fusion [14]. Plasmid DNA was purified with the QIAprep Spin Miniprep (QIAGEN), verified by full insert sequencing and introduced into the L40ΔGal4 (*MATa*) bait yeast strain [15]. Alternatively, prey fragments were directly extracted from the prey plasmid and subsequently cloned in pB27 to use them as secondary or tertiary baits.

The prey library in yeast was constructed from adult (Ambion AM7983) and fetal (Stratagene #778020) human skeletal muscle poly(A) RNA. Random-primed cDNA fragments were prepared from these two RNA pools and cloned in the pP6 plasmid derived from the original pACT2 (Clontech) as a C-terminal fusion of the Gal4 transcription activating domain. Altogether, 90% of the plasmids contained a cDNA insert with an average size of 600 bp. After amplification in *Escherichia coli* (50-100 million independent clones), the Y187 (*MATalpha*) yeast strain was transformed with an equimolar pool of the adult and fetal cDNA libraries. Ten million independent yeast colonies were then collected, pooled and stored at -80°C as equivalent aliquot fractions of the same prey library. Validation of the prey library was performed by recapitulating several published interactions as described in [14]. Bait proteins belonging to different functional classes were used: a GTPase (Rac1), a transcription factor (TP53), a splicing factor (SF1) and a component of a E3-ligase complex (BTRC).

### Screening procedure and identification of prey fragments

Y2H screens were performed using a mating method as described in [14] at the Hybrigenics facility (Hybrigenics, Paris). As first step, small-scale screenings were performed to assess toxicity and auto-activation capacity of the baits and to adjust selective pressure of the screens accordingly. In particular, the optimal concentration of 3-aminotriazol (3-AT) was determined prior to performing each large-scale screen. Auto-activating baits able to activate transcription of the reporter gene by themselves were identified and were not considered for large-scale screenings. Subsequently, each bait clone was tested in a full-size screen against an average of 103 million yeast prey clones, equivalent to ten-fold coverage of the library. All positive clones were picked and the corresponding prey fragments were PCR-amplified and sequenced at their 5^′^ and 3^′^ junctions. Sequence contigs were built and identified by comparison to the NCBI Human RefSeq database as described in [14].

### Identification of interacting fragments and scoring of the interactions

Following contig assembly of positive clones, the common sequence shared by the assembled prey fragments was used to define the SID along each prey protein. Furthermore, for each interaction, a PBS was computed with E-values ranging from 0 to 1 to establish six distinct categories: PBS-A to -E (see [14] for details on calculation). The technically most reliable interactions were associated with the PBS-A, -B or -C categories (with P values < 1^*e*-10^ for PBS-A;< 1 ^*e*-5^ for PBS-B and < 1^*e*-2.5^ for PBS-C) and are found in two reciprocal and independent screens (X->Y and Y->X) and/or in interaction cycles (X-Y, Y-Z and X-Z) and/or in a single screen but with many overlapping prey fragments. Interactions were assigned to the PBS-D category when they were supported either by a single experimental clone from a screen or by several clones bearing the same start and stop positions, the SID being identified by a singleton fragment instead of a family of several overlapping fragments. This PBS-D category corresponds to a heterogeneous group of interactions that theoretically could consist of technical false-positive interactions as well as true-positive interactions hardly detectable by Y2H systems (due to constraints in tri-dimensional conformation of bait or prey domains, toxicity in yeast, poor mRNA representation of the prey in the library, …). All the PBS-D should therefore be considered as putative unless validated by a second technique. The PBS-E category characterizes SID that have been found as prey in more than ten independent screens with unrelated bait proteins in all screenings performed with human libraries at the Hybrigenics facility. These interactions potentially represent possible false-positives of the Y2H system as well as interactions with proteins known to be highly connected due to their biological function or with proteins containing a biochemically promiscuous motif. Finally, interactions with proteins or domains corresponding to known false positives of the Y2H system as it is described above were removed from the data and from our analyses. Examples of yeast growth assays describing interactions with the different PBS categories using the same experimental procedures can be found in [16-18].

### Antibodies

The antibodies used for immunoprecipitation of the baits are BD Biosciences anti-TCAP (T26820-050), Novocastra Laboratories Ltd anti-DYSF (NCL-Hamlet) and Santa Cruz Biotechnology anti-ABI1 (sc-30038), anti-ACTN2 (sc-15335), anti-DES (sc-14026), anti-MYOM1 (sc-30390) and anti-TCAP (sc-8725).

The antibodies used for prey detection by western blot are Abcam anti-SNAPIN (ab37496), Abnova anti-ADPGK (H00083440-M01), anti-APPL1 (H00026060-A01) and anti-ENO1 (H00002023-M04), Aviva anti-KBTBD10 (ARP38732_T100) and Santa Cruz Biotechnology anti-KIF1B (sc28540) and anti-KTN1 (sc33562).

The antibodies used for immunochemistry and Duolink assays and their corresponding dilutions are: Abcam anti-CMYA5 (ab75351, 1:50) and anti-OPTN (ab23666, 1:100), Abgent anti-DGKD (AP8126b, 1:50), Abnova anti-DNAJB6 (H00010049-M01, 1:100) and anti-EEF1G (H00001937-M01, 1:50), Novocastra Laboratories Ltd anti-DYSF (NCL-Hamlet, 1:20), Proteintech Group anti-SNAPIN (10055-1 AP, 1:50), Santa Cruz Biotechnology anti-ACTN2 (sc-15335, 1:100), anti-ALMS1 (sc-54507, 1:50), anti-APPL1 (sc-67402, 1:50), anti-DES (sc-14026, 1:100), anti-FLNC (sc-48495, 1:100), anti-KIF1B (sc-28540, 1:50), anti-MYOM1 (sc-30390, 1:100), anti-MYOM2 (sc-50435, 1:200) and anti-NEB (sc-28286, 1:100) and Sigma anti-NPHP3 (HPA009150, 1:75).

### Co-immunoprecipitation

The bait proteins were isolated from R9 cell extracts (a gift from Dr. Anne Galy, Inserm U790, Evry, France) at myoblast or myotube stage (7 to 10 days of differentiation) or from gastrocnemius muscle excised from four week-old mice and homogenized in 6 ml lysis buffer (Tris 20 mM, pH 7.5, NaCl 50 mM, EGTA 2 mM, Triton 1%, Protease Inhibitor Cocktail (Complete mini, Roche), E64 2 μM) using a FastPrep-24 apparatus (MP Biomedicals). The mouse samples correspond to a protocol approved by Genethon’s ethics committee under the number CE11_014 and performed in accordance with the directive of 24 November 1986 (86/609/EEC) of the Council of the European Communities. After centrifugation of the lysates, 500 μg to 1 mg of proteins in 1 ml were incubated with 30 μl of protein G–Sepharose beads (Amersham) for 1 h to clear from nonspecific binders. The protein extract was then subjected to immunoprecipitation by 1 h incubation at +4°C with 2 to 4 μg of primary antibodies corresponding to the baits, then 30 μl of protein G Sepharose beads (Amersham) were added and incubation was carried out for 2 h or overnight at +4°C.

After centrifugation at 1000 g for 5 min, the immunocomplex was washed three times with 1 ml of buffer and resuspended in 15 μl 4x NuPAGE LDS sample buffer (Invitrogen) and dithiothreitol reducing agent. Samples were then heated at +70°C for 10 min and centrifuged briefly. Protein complexes were separated by electrophoresis on SDS-PAGE NUPAGE 4-12% Bis-Tris gel (Invitrogen). Transfer of the proteins was performed on PVDF membrane and verified by staining with Ponceau red. Immunostainings were performed with primary antibodies corresponding to prey and IRD-680 or 800 donkey anti-mouse, -rabbit or -goat as secondary antibodies according to LI-COR’s protocol. Bands were then visualized with the Odyssey infra-red imaging system (LI-COR-Biosciences) at 700 nm (red) and 800 nm (green).

### Immunohistochemistry

Indirect immunofluorescence microscopy assays were carried out on transversal cryosections prepared from normal human paravertebral striated muscles of a 13-year old female biopsy obtained from the biobank Myobank under the validation number AC-2008-87 from the French ministry of research (Institute of Myology, Paris). The sample was treated anonymously. Frozen slides were air-dried for 30 min at room temperature, fixed with 4% PAF for 5 min, washed 3 × 5 min in PBS, incubated in a blocking buffer (4% BSA, 0.02% Triton) for 30 min, washed in PBS, then incubated with a biotin blocking solution (Vector Laboratories, SP-2001) for 15 min and washed in PBS for 5 min. Slides were stained at room temperature for 1 h or at +4°C overnight with primary antibodies diluted in the labeling solution (1% BSA / PBS). Slides were then incubated with a donkey anti-mouse-Alexa 488 for dysferlin and a donkey (anti-rabbit or anti-goat) biotinylated secondary antibody for its partner (dilution 1:1000) for 45 min, washed 3x 5 min in PBS and stained with streptavidin coupled to Alexa-594 (Molecular Probes, dilution 1:500 in PBS) for 30 min. For nucleic acid staining, slides were then incubated with TOPRO-3 (Molecular Probes, dilution 1:2000) for 5 min, washed 2 x 5 min in PBS and 1x in water for 2 min. Slides were subsequently mounted in Fluoromount-G™ (SouthernBiotech, 0100-01). Images were acquired using the 40x or 63x objective of a Zeiss Axiovert 100 M. LSM.510 Meta laser scanning confocal microscope and the constructer software. Colocalization analyses were performed by statistical analysis of the correlation between the intensity values of red and green pixels in a dual- channel image. The JACop plug-in [19] for ImageJ (Rasband, W.S., ImageJ, U. S. National Institutes of Health, Bethesda, Maryland, USA, http://imagej.nih.gov/ij/, 1997-2011 ) was used to calculate Pearson’s Correlation coefficient. Co-localization was defined as strong for 0.5<R≤1, medium for 0.25<R≤0.5 and low for R≤0.25.

### Proximity ligation assays

The Duolink® kit (Olink Bioscience) is based on the use of two unique and bi-functional probes called PLA™, each probe consisting of a secondary antibody attached to a unique synthetic oligonucleotide that acts as a reporter. After a 10 min fixation with paraformaldehyde 4% and blocking (BSA 5% in PBS) steps, muscle sections were stained with one or two primary antibodies depending on the experiment (single protein detection or detection of interacting proteins) over-night at +4°C. After washing, the sections were incubated with the secondary oligonucleotide-linked antibodies (PLA probes) provided in the kit. The oligonucleotides bound to the antibodies were hybridized, ligated, amplified, and detected using a fluorescent probe (Detection Kit 563). Dots were detected with the Zeiss laser scanning confocal microscope and intensity signal counted using ImageJ software ( http://imagej.nih.gov/ij/). A series of controls were performed for each analysis (bait antibody only, prey antibody only and negative control for which the primary antibody is omitted).

For quantification analysis: three images were acquired under the same conditions (laser power, PMT gain and pinhole) for each experiment. For each image, five fibers were randomly selected and used to count all positive spots within each compartment (total of 15 cells). The regions of interest (ROI) for membrane and cytoplasm compartments were separately delimitated manually and signal quantification was performed on all identified spots using the ImageJ software. For each compartment, we considered that the PPI was validated by the assay when the mean signal ratio between the PLA images of the PPI, “PPI signal”, and the control images of the prey, “PREY signal”, was superior to 0.2, indicating that the interaction with the prey potentially recruited more than 20% of the interacting partner in the delimited compartment.

### Bioinformatics and statistical analyses

IpScan [20] with Interpro 17.0 [21] was used to annotate the protein sequences. The SID coordinates were compared with the position of the different Interpro domains. Cytoscape tools ( http://www.cytoscape.org) were used to infer connectivity, a parameter that indicates the number of proteins that directly interacts with a given protein. Comparison of PPIs identified by our Y2H screenings with previously published PPIs was performed using the iRefWeb interface ( [22]; http://wodaklab.org/iRefWeb/) by considering direct interaction found in mammals.

GO mapping and clustering were performed with the DAVID 6.7 web interface [23,24] using the Functional Annotation Clustering tool and the GOTERM_FAT annotation categories in order to filter the broadest GO terms. For the LGMD-centered dataset, a list of official gene symbols was used to identify the proteins and within each identified GO cluster, GO terms were analyzed in terms of hierarchy to identify the most specialized children terms common to all proteins within the cluster and these terms were reported as “shared GO” annotations (Additional file 1: Table S1). To analyze whether our datasets were statistically different from a random dataset, GO clustering was also performed with a list of Uniprot accessions for the 19220 human protein-coding genes (HGNC, http://www.genenames.org/). The number (Shared-i) of human proteins with which a given bait protein (Bait-I) shared a GO cluster was calculated for all three GO classes (BP, MF and CC) and all baits and the number of protein pairs not sharing a GO cluster was deduced (NonShared-i = 19220 - Pi). The overall frequency of expected shared and non shared protein pairs was calculated as the ratio between the sum of Shared-i and the sum of all pairs (76 × 19220), and the sum of NonShared-i and the sum of all pairs, respectively. A Chi-2 test (P<0.05) was used to compare expected values with observed values from the LGMD-centred dataset or the subset consisting of all PBS-A to -C categories.

GO enrichment analyses were performed using the DAVID Functional Annotation tool with Uniprot accession numbers as identifiers, the *Homo sapiens* background and the GOTERM_FAT annotation categories. Enrichment at 1% significance level was defined with a modified Fisher exact *P* value (the “EASE” score) as recommended by the DAVID interface.

Statistical analysis of obtained proportions for the other analyses was done using the Fisher test function in R.

## Results

### Bait design and screening procedure

The procedure for choosing the bait protein sequences to be used as baits for our Y2H screenings lied in three successive steps in which primary, secondary and tertiary baits were selected to perform three rounds of Y2H screenings. First, we selected nine proteins involved in recessive LGMD forms and four proteins that were either known as LGMD-binding proteins or described as having a role in muscular atrophic processes (Table 1). Design of the baits excluded hydrophobic trans-membrane domains, signal peptides and transcriptional trans-activation domains to ensure the best Y2H screening conditions. We chose either full-length coding sequences or specific domains as bait, especially, for large proteins such as titin (TTN) and dysferlin (DYSF). Overall, we selected 20 primary bait domains.

**Table 1:** A/Description of primary baits

**Protein name**	**Protein symbol and LGMD form**	Bait domain coordinates (aa)*	**Bait domain description**
calpain 3	CAPN3 (LGMD2A)	T417-S643	C2-like domain + exons15-16
		M1-A822	full length protein with the C129S mutation in the autocatalytic site
dysferlin	DYSF (LGMD2B)	L2-I485	N-terminal DYSF regions containing the first three C2 domains
		Q851-D1200	central DYSF domain
		I1145-L2026	C-terminal DYSF domain containing the last four C2 domains and excluding the transmembrane span
γ- sarcoglycan	SGCG (LGMD2C)	M1-L36	cytoplasmic domain
α-sarcoglycan	SGCA (LGMD2D)	M313-H388	cytoplasmic domain
β-sarcoglycan	SGCB (LGMD2E)	E10-A65	cytoplasmic domain
δ-sarcoglycan	SGCD (LGMD2F)	E1-Y36	cytoplasmic domain
titin-cap (telethonin)	TCAP (LGMD2G)	M1-G167	full length protein
tripartite motif-containing 32	TRIM32 (LGMD2H)	L66-P654	full length protein minus the RING domain
titin	TTN (LGMD2J)	V97-K469	exons 4-8 from the Z-Disc region
		I741-G948	exons 14-17 from the Z-Disc region
		R2120-L2564	exons 28-33 from the Z-Disc region
		A8831-E9158	exons 108-114 from the N2A-PEVK region
		T32840-I33423	exons 358-363 from the Mline region
ankyrin repeat domain 1	ANKRD1	M1-F319	full length protein
ezrin	EZR	M1-S536	full length protein minus the actin-binding C-terminal domain
F-box protein 32 (MAFbx)	FBXO32	M1-F356	full length protein
tripartite motif-containing 63 (MuRF1)	TRIM63	M1-Q354	full length protein

* amino acid coordinates refer to the translated products of the nucleotide sequences indicated in Additional file 2: Table S2.

For each bait domain, we first assessed its toxicity and auto-activation capacity by a small-scale Y2H screen and then performed a large-scale Y2H assay by screening a high-complexity cDNA prey library obtained by random priming of poly(A)+ RNA from adult and fetal human skeletal muscles that we constructed for this purpose. The bait interaction was tested against an average of 103 million prey clones to insure a ten-fold coverage of the prey library. Positive prey clones were sequenced and compared to the NCBI human RefSeq database for prey identification. Contig assembly of positive clones was performed to isolate the minimum interacting domain(s) on each prey sequence [Selected Interacting Domains (SID)]. We used clone coverage and local topology information to compute a confidence score [Predicted Biological Score (PBS)] and classify each PPI into five categories: PBS-A, -B or -C for the most reliable interactions, PBS-D for putative interactions involving a single bait clone and PBS-E for interactions involving highly connected proteins.

We then examined the interaction networks resulting from the first screenings according to the PBS categories and literature data and conducted selection of secondary and tertiary baits. First, we isolated 54 prey proteins of interest to design 57 new bait domains for a second round of screenings and then, we used the resulting Y2H network to select 10 additional proteins corresponding to 11 baits for a third and last round of screening. Two of the chosen baits showed autoactivation capacities (*CMYA5*_Q3501-K4069_ and *RCOR3*_M1-L296_ fragments) and were therefore discarded. Overall, we successfully carried out 87 large-scale Y2H screenings using 76 different bait proteins. The comprehensive set of baits is listed in Additional file 2: Table S2.

### General properties of the Y2H interaction map

The 87 screens led to the identification of 1625 SIDs. On average, each Y2H assay yielded 18.7 SIDs with a range of 1 to 107. This corresponds to a mean of 19.7 PPIs per bait protein with 53 pairs of connected proteins showing two or more SIDs on the prey protein (Table 2). Performing secondary and tertiary screenings theoretically allowed us to identify reciprocal hits between the secondary/tertiary bait protein and the protein it was originally found to interact with. Practically, reciprocal screenings were not symmetrical because the prey and bait domains were expressed as fusion with a DNA-binding or GAL4-activating domain, respectively and because domains first identified as prey, and then chosen as bait, were rarely identical for practical cloning reasons. Nevertheless, we were able to reciprocally detect nine interactions (Additional file 1: Table S1) that were automatically grouped into the PBS-A category.

**Table 2:** General features of the LGMD-centered interaction map

	**Value**	**Range, percentage**
Total number of bait domains/proteins	87 / 76	
Average number of tested diploids	103 10^6^ per screen	20 10^6^ - 203 10^6^
Average number of processed positive clones	155 per bait domain	3-686
Total number of proteins/PPIs	1018/1492	
PBS-A	234	15.7%
PBS-B	147	9.8%
PBS-C	110	7.4%
PBS-D	800	53.6%
PBS-E	201	13.5%
Average number of SIDs per bait domain	18.7	1-107
Average number of partners per prey protein	1.5	1-28
Mean connectivity	2.88	
Total number/average size of identified SIDs	1625 /231aa	

**Figure 1:**
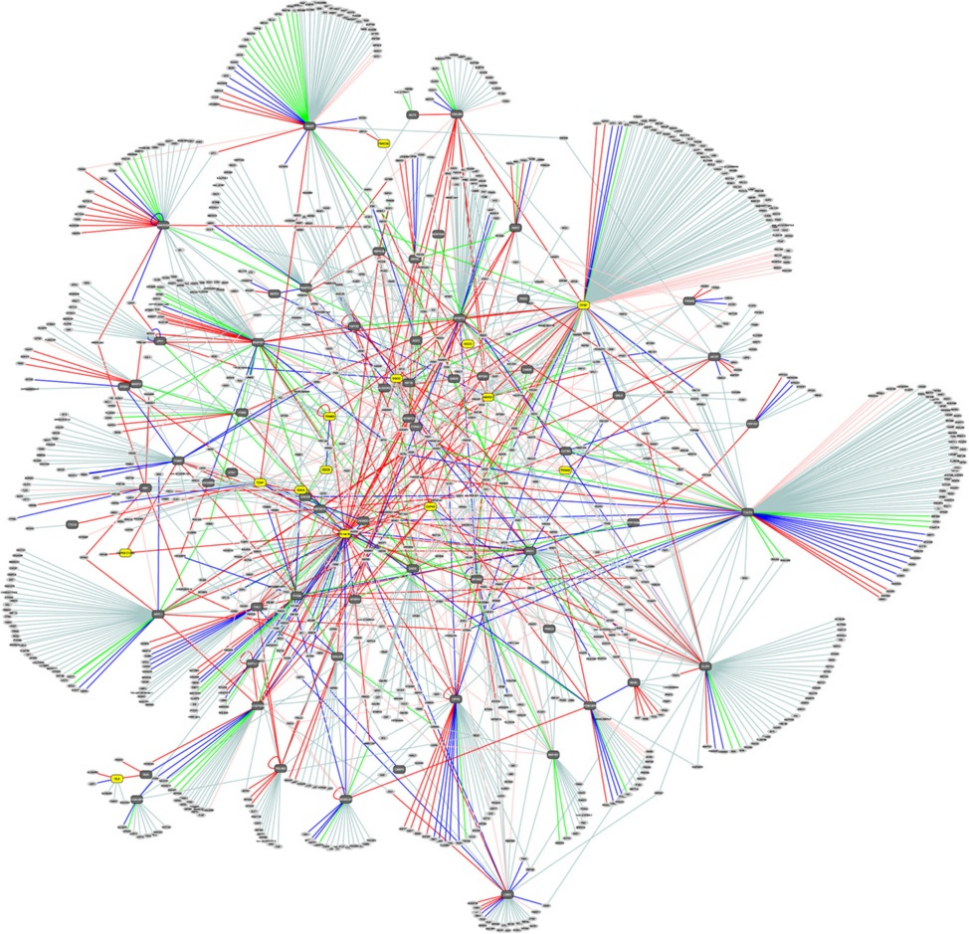
**The LGMD-centered network.** A total of 87 Y2H screens was performed starting from the LGMD proteins. Results from all individual Y2H screenings were assembled in a single network of 1492 PPIs connecting 1018 proteins. Primary baits are depicted as yellow rectangles. Secondary and tertiary baits are depicted as grey octagons. Preys are depicted as white circles. Interactions between pairs of proteins (nodes) are depicted by edges with colors according to the PBS category (PBS-A: red, PBS-B: dark blue, PBS-C: green, PBS-D: light blue, PBS-E: light pink). To visualize details on the image, readers are invited to zoom in.

Results from all individual Y2H screenings were assembled in a single network of 1492 PPIs connecting 1018 proteins (LGMD-centered network, Figure 1). Topological analysis of this network using the Cytoscape platform ( http://www.cytoscape.org) revealed an average connectivity of 2.88 partners per connected protein. The mean shortest path length between any of two proteins was calculated as 3.75. The set of interactions presenting the highest level of confidence consists in the PPIs ranked into the PBS-A, -B or -C categories and comprises 32.9% of all PPIs, a figure similar to the one found by [14]. This set defined the most probable true-positive interactions and comprised 491 PPIs consisting of 376 interacting proteins. Among the other remaining interactions, 53.6% and 13.5% were classified in the PBS-D and -E categories, respectively. The high level of PBS-D interactions reflects the high complexity of the screened library and the fact that it is screened to saturation but should be considered as putative. General properties of the LGMD-centered interaction map are summarized in Table 2 and the complete list of PPIs along with their PBS category is available in Additional file 1: Table S1.

### Examination of interaction domains

Contrasting with other Y2H approaches in which bait proteins are tested against full-length prey libraries [1,2], our approach was domain-oriented since the prey library was generated as random-primed cDNA with an average fragment size of 800-1000 bp and since some of the baits consisted of selected domains. For each prey protein involved in a PPI ranked in the PBS-A, -B or –C categories, we analysed its SID(s) and compared their coordinates with predicted InterPro domains to detect SIDs that were included in or included a documented domain. In total, 601 SIDs were computed and showed an average size of 176 aa (Additional file 3: Table S3). This analysis resulted in the identification of 311 distinct SIDs that could be unambiguously associated with one or more InterPro domains either because they were fully included within one such domain (82 SIDs) or because they fully included one or several of them (248 SIDs). The most frequent InterPro domains found within SIDs (in more than 4% of the SIDs; Table 3) were Immunoglobulin-like domains (IPR003599; IPR003598 and IPR007110), Zinc Finger C2H2 (IPR007087), Fibronectin type III domain (IPR003961), Ankyrin repeat (IPR002110) and Nebulin motif (IPR000900). Finally, we compared the occurrence of InterPro domains fully included within the set of analyzed SIDs with their occurrence in the full human proteome according to [25]. Interestingly, three among the five top-ranking domains in Human were also present with a high frequency in the analyzed dataset although with different ranks and percentages (Table 3). The other two most frequent domains in our subset (Ankyrin repeat and Nebulin motif) were largely over-represented (p-value = 7.62e-16 and <2.81e-20, respectively) compared to the global human proteome whereas the three and four top-ranking domains in Human (EGF/laminin and P-loop nucleotide triphosphate hydrolase) were scarce in the analyzed SIDs.

**Table 3:** Most frequent domains in the LGMD-centered dataset and representativeness in the human proteome

**Domains**	**% in the total of SID**	**% in the human proteome (according to Müller et al., Genome Res 2002)**
Immunoglobulin (IPR003599; IPR003598; IPR007110)	22.5% (n=141)	4.2% (n=1214; r=2)
Zinc finger, C2H2-type (IPR007087)	12.8% (n=80)	17.6% (n=5092; r=1)
Fibronectin type III (IPR003961)	9.63% (n=60)	2.9% (n=842; r=5)
Ankyrin repeat (IPR002110)	5.3% (n=33)	0.9% (n=278; r=14)
Nebulin 35 residue motif (IPR000900)	4.5% (n=28)	n.i.

(n.i.= not indicated; n= number of occurrences; r= rank in the human proteome).

### Quality assessment of the Y2H network

For all large-scale studies aimed at experimentally identifying molecular interactions, the technical false positive rate is of special concern. We addressed this problem with the assignment of a PBS category. Furthermore, literature mining, cross-validation assays, and functional correlations were used to further estimate the overall solidity of the network.

#### Comparison with known interactions data

We first compared our LGMD-centered Y2H dataset with literature-curated interactions between the corresponding mammalian proteins that are referenced in the molecular interaction database iRefWeb ( http://wodaklab.org/iRefWeb/[22]). This web interface reports data on PPIs consolidated from major public databases such as IntAct, BIND or HPRD. Among our PPIs, 72 were already reported, representing 4.8% of our map (Additional file 1: Table S1), a figure slightly above others found in large-scale Y2H studies aimed at exploring the human interactome (3.4% [2] and 3.8% [6]). We observed a strong enrichment for literature-based interactions within the PBS-A category for which 18% of PPI (43) correspond to previously known interactions (p-value = 1.54e-18), thus confirming the correlation between the PBS and the biological significance of the interaction.

#### Experimental cross-validations

**Figure 2:**
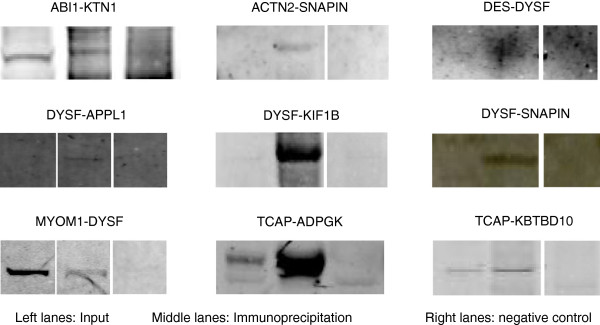
**Immunoprecipitation analysis of a subset of interactions.** A subset of interactions from the baits: abl-interactor 1 (ABI1), DYSF, SNAP-associated protein (SNAPIN) and telethonin (TCAP) was assessed using co-immunoprecipitation using myogenic cells or in gastrocnemius mouse muscle. Immunoblotting was performed with anti-prey antibody. Left lanes= input sample. Middle lanes: Protein lysates co-immunoprecipitated with anti-bait antibody. Right lanes= negative control where the primary antibody was omitted. One PPI for ABI1, 5 for DYSF, 1 for SNAPIN and 2 for TCAP were confirmed as positive by the co-immunoprecipitation assays.

To provide more evidence that the identified physical interactions are genuine, we applied different experimental techniques that enable detection and visualization of protein interaction. We first tested a subset of PPIs for the baits: abl-interactor 1 (ABI1), DYSF, enolase 1 (ENO1),SNAP-associated protein (SNAPIN) and telethonin (TCAP), using co-immunoprecipitation in murine R9 myogenic cells at myoblast or myotube stages or in gastrocnemius mouse muscle. A large proportion of the 42 interactions tested were technically inconclusive due in part to the quality of the available antibodies or stickiness of both proteins. Among the 14 technically conclusive, 9 (64%) were confirmed as positive by the co-immunoprecipitation assays (1 PPI for ABI1, 5 for DYSF, 1 for SNAPIN and 2 for TCAP; Figure 2 and Additional file 1: Table S1). Several interesting outcomes emerge from these validations that reinforce and extend the knowledge we have on LGMD proteins or open new directions for specific areas of research. For example, the interaction between DYSF, the LGMD2B protein, and SNAPIN, a component of the SNARE complex that is required for vesicle fusion [26] is appealing with regard to the role of DYSF in membrane repair [27]. The positive interaction between kinectin (KTN1), a kinesin anchoring protein that modulates the endoplasmic reticulum interaction with the microtubule and that is known to interact with Rho GTPases [28], and ABI1, an adaptor protein involved in the transduction of signals from Ras to Rac and the regulation of actin polymerization [29], suggests a role for this interaction in the regulation of the muscle cytoskeletal remodeling.

**Figure 3:**
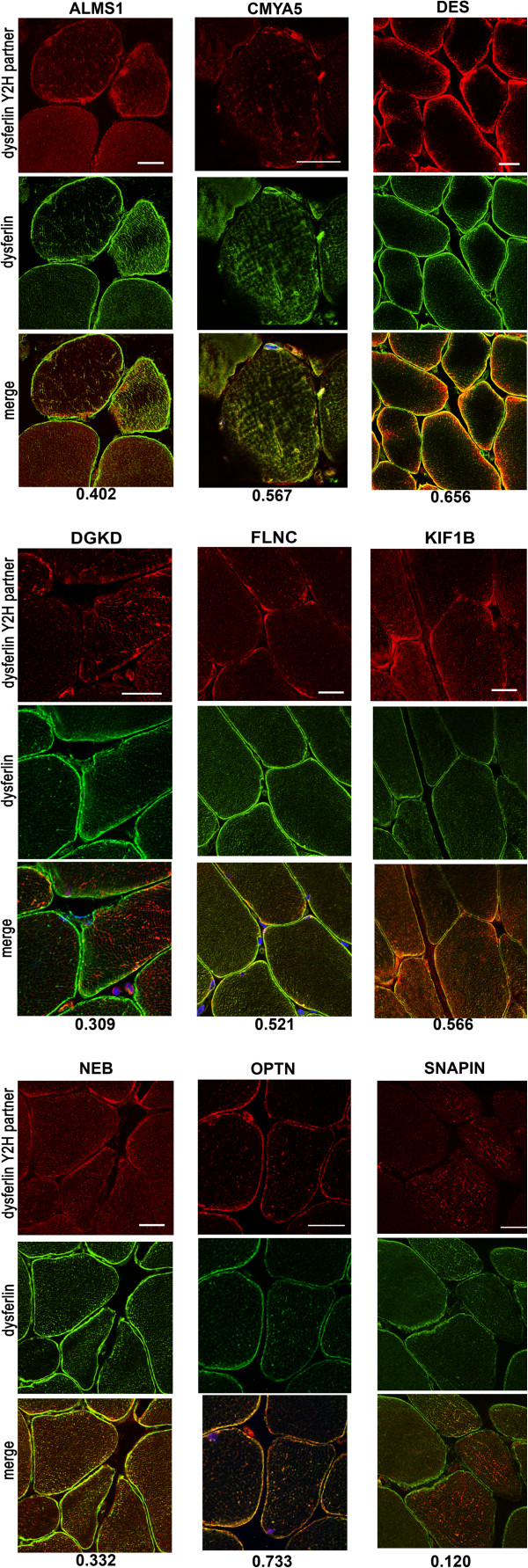
**Co-localization analyses of dysferlin and its partners.** Double immunostaining for DYSF (Alexa488, green) and its Y2H partners (Alexa594, red) in cross human muscle cryosections by alphabetic order. The images were taken with a 40x objective for DES, FLNC, and NEB and a 63x for OPTN, KIF1B, DGKD, SNAPIN, and CMYA5. Scale bars = 20 μm. The Pearson’s coefficient is indicated under the merge image for each PPI. As expected, DYSF showed a membrane-associated pattern and a reticular cytoplasmic pattern corresponding to T-tubules on transversal section. Depending on the tested protein, the partner pattern is variable.

We then investigated intracellular co-localization on normal human muscle sections of one particular bait, DYSF, with its Y2H partners for which quality antibody was available for immuno-fluorescence assays. Among the 14 PPIs investigated, five showed no apparent co-localization while the others demonstrated different degrees of co-localization at the sarcolemma and/or in the cytoplasm (Figure 3). Analysis of Pearson’s coefficient showed that DYSF labeling presented a strong correlation with the cardiomyopathy-associated protein 5 (CMYA5, 0.567), DES (0.656), filamin-C (FLNC, 0.521), kinesin family member 1B (KIF1B, 0.566) and optineurin (OPTN, 0.733), a medium correlation with Alstrom syndrome protein 1 (ALMS1, 0.402), diacylglycerol kinase delta (DGKD, 0.309), and nebulin (NEB, 0.332) and a low correlation with SNAPIN (0.12).

**Figure 4:**
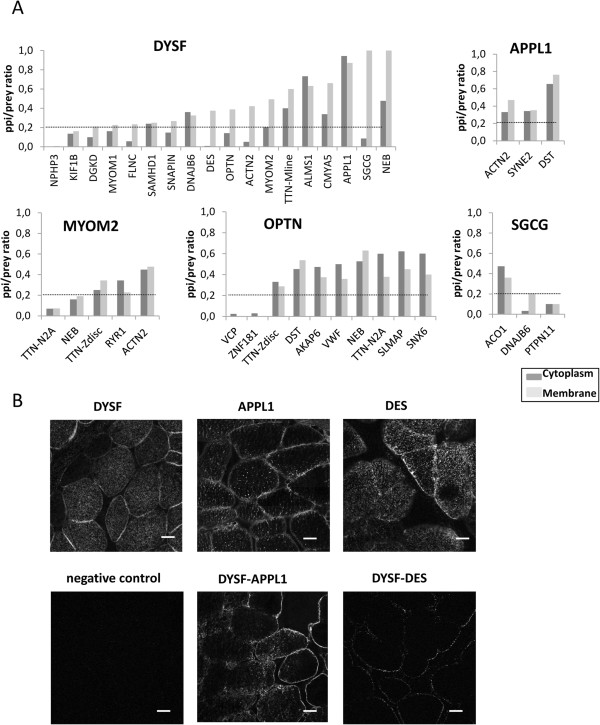
**Proximity ligation assays of a subset of interactions. A/** Graphs showing the ratio of the signal for the PPI divided by the signal for the prey protein alone with respect to the ROI corresponding to membrane (light grey) or cytoplasm (dark grey) for the bait proteins DYSF, APPL1, MYOM2, OPTN and SGCG. Prey proteins are named with their gene symbol. The line corresponding to 20% is indicated for each graph. For technical reasons, in the case of DYSF/SGCG and MYOM2/TTN interactions, the PPI/prey ratio is calculated as the ratio of SGCG and TTN molecules with respect to DYSF and MYOM2 although DYSF and MYOM2 were found as preys of SGCG and TTN baits. For MYOM2 and OPTN, two TTN antibodies were tested with epitopes at the Z-disc and N2A regions, respectively. Among the 44 tested interactions, 34 showed a “PPI signal” with various proportions between membrane and cytoplasm. **B/** Representative confocal images of PLA results. Duolink amplifications are visualized by fluorescence (white dots). Upper panels: PLA+/- labeling of single proteins. Left panel = negative control consisting of DYSF with probes corresponding to the PPI for evaluation of background staining. Middle panel: the DYSF-APPL1 interaction signal showed a strong labeling both at the membrane and the cytoplasm. Right panel: the DYSF-DES labeling showed that the signal is mainly located at the membrane. Scale bars= 20 μm.

We also visualized the sub-cellular localization of the interactions using a Duolink proximity ligation assay (PLA) for 44 PPIs. The Duolink technology is a combination of immunohistochemistry and rolling circle amplification and is based on the use of bifunctional probes consisting of a secondary antibody attached to a synthetic oligonucleotide. It generates a quantifiable signal indicative of close proximity (<40 nm) between two antigens. For our purpose, primary antibodies against five different baits [DYSF, OPTN, γ-sarcoglycan (SGCG), adaptor protein containing PH domain, PTB domain and leucine zipper motif 1 (APPL1) and myomesin2 (MYOM2)] and some of their partners identified in our Y2H screenings (18 interacting partners for DYSF, 10 for OPTN, 3 for SGCG, 3 for APPL1 and 5 for MYOM2) were used to obtain a PLA “PPI signal”. In a separate experiment, only the primary antibody of the prey was used to generate the “Prey signal”. In this way, the cellular site(s) where the binary interactions were occurring could be visualized. Among the 44 tested interactions, 34 showed a “PPI signal” with various proportions between membrane and cytoplasm. An image analysis was performed to calculate the ratio of the “PPI signal” compared to the “Prey signal” separately at the membrane or cytoplasm locations (Figure 4A). Two examples of DYSF interactions are depicted in Figure 4B. The single protein detection of DYSF revealed as expected the presence of the protein at the membrane and at the T-tubule network within the cell (Figure 4B). The APPL1 protein seems almost entirely engaged in the interaction with DYSF both at the membrane and cytoplasm locations. In contrast, DES/DYSF association was visualized only at membrane level.

In total and among the 54 PPIs investigated using these three different techniques, 40 interactions were considered as relevant by at least one technique (74%) with 20 of them from the PBS-A, -B, or -C categories and the others from the PBS-D category. The 26% remaining interactions identified by our Y2H screens that could not be experimentally cross-validated in our hands consist in 3, 3, 2 and 5 interactions classified in the PBS-A to –D categories, respectively.

#### Examination of GO annotations

**Figure 5:**
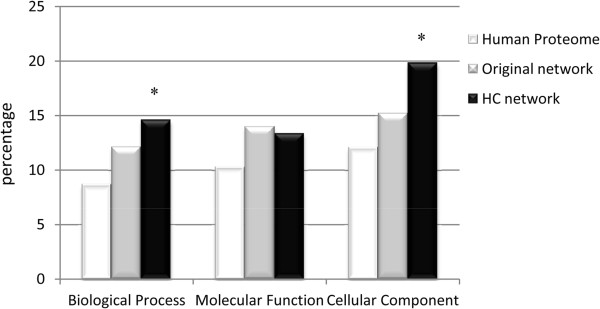
**Fraction of proteins pairs sharing a GO annotation cluster.** Each bar represents the fraction of proteins pairs that share a cluster within each of the three GO categories (biological processes, cell components or molecular functions; percentage indicated). Asterisks above the bars indicate an observed value statistically different from the one expected in the Human proteome dataset (Chi-2 test, P<0.05).

We performed computational analyses using the Database for Annotation, Visualization, and Integrated Discovery (DAVID) web-resource ( http://david.abcc.ncifcrf.gov/) to detect pairs of interacting proteins known to participate in similar biological processes (BP), to be part of the same cell components (CC) or to perform similar molecular functions (MF). In the LGMD-centered network, 84, 72 and 82% of proteins were annotated for the BP, CC and MF GO categories, respectively. Among the annotated proteins, we identified 449 pairs of proteins (30%) that share a GO annotation, including 168 pairs within the PBS-A, -B, or -C categories (34%; Additional file 1: Table S1). More precisely, among PPIs from the PBS-A, -B and -C categories, a significantly higher number of PPIs with shared BP or CC GO annotations compared to the Human proteome were detected (Chi-2 test, P<0.05, Figure 5), confirming again the correlation between the PBS and the biological significance of the interaction.

### The NMD proteins in the LGMD-centered network

We examined our LGMD-centered interactome map to pinpoint interactions involving proteins identified as the genetic cause of one or more hereditary NMDs by searching the OMIM database. We identified 199 proteins of the network that are associated with human monogenic diseases including 77 proteins whose defects have been described as the genetic cause of one or more hereditary NMD (Additional file 4: Table S4) . Among this last group, 43 proteins correspond to myopathies including the recently described DNAJB6 [30], 20 to cardiomyopathies, 17 to neuropathies, 8 to metabolic muscle diseases, 3 to excitation abnormalities and 8 to unclassified NMD. From these 199 proteins, it was possible to construct a protein interaction network including 88 proteins in 113 interactions (Additional file 5: Figure S1). No specific repartition of NMD and non-NMD proteins was noticed.

**Figure 6:**
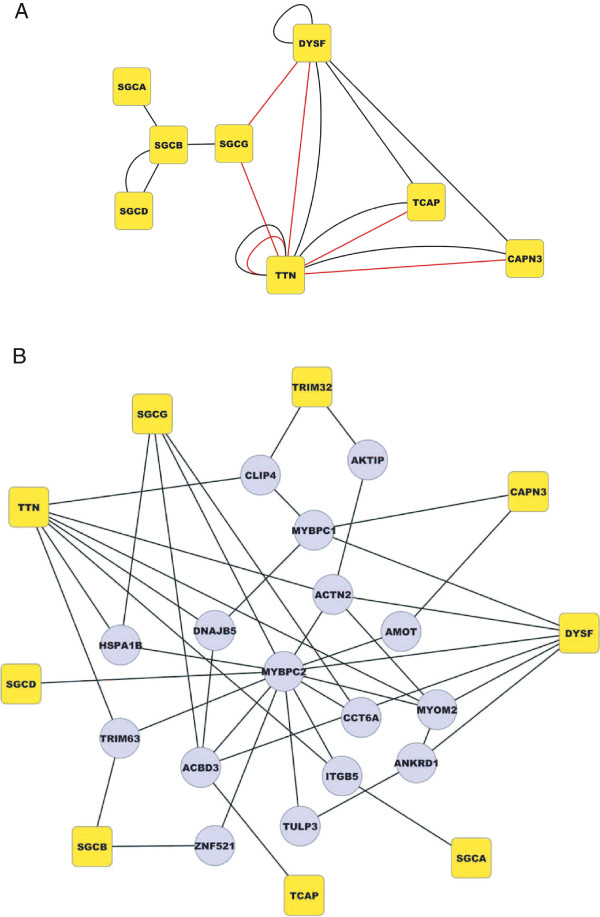
**The network linking the LGMD proteins. A/** Interactions between pairs of LGMD proteins. Interactions are depicted by black lines if previously reported and in red if detected in our work for the first time. **B/** Subnetwork presenting links of three nodes or shorter with LGMD proteins. LGMD2 proteins and their preys are depicted as yellow and grey nodes, respectively. Only preys that show at least two interactions with LGMD proteins are presented. The figure indicates the existence of a dense network around the group of LGMD-causing proteins.

We then examined the sub-network consisting of all the LGMD and other known NMD-causing proteins, and their interacting partners. This sub-network contains 56 proteins whose defects have been described as the genetic cause of one or more hereditary NMD. Aside from the nine LGMD proteins used as baits in our screenings, this network contains three additional LGMD proteins, plectin (LGMD2M), myotillin (LGMD1A) and lamin A/C (LGMD1B). Thirty-five connections of LGMD-proteins with other NMD-causing proteins were identified with 28 novel and direct interactions. In particular, this analysis revealed three novel direct interactions linking TTN, DYSF and SGCG (Figure 6A). We examined the type of diseases the non-LGMD proteins are responsible for and found that proteins involved in congenital and metabolic myopathies as well as other neuromuscular disorders were significantly over-represented (p-value= 1.04e-05, 0.025 and 0.040, respectively). It should be noted that the proteins involved in the metabolic myopathies glycogen storage disease type 13 (GSD13) and glycogen storage disease type 5 (GSD5), that are β-enolase (ENO3) and the muscle form of glycogen phosphorylase (PYGM), respectively, present a high number of physical interactions with the LGMD proteins group. Interestingly, we observed the existence of a dense network around the group of LGMD-causing proteins with many of them linked by paths of three nodes or shorter (Figure 6B). It can be noted that among the hub proteins in this network are proteins located at three important positions of the sarcomere: actinin α2 (ACTN2) for the Z-disc, myosin-binding protein C, slow-type (MYBPC1) and MYBPC2 for the N2A-line and MYOM2 for the M-line.

Considering that the LGMD-centered interactome is strongly enriched in known NMD-causing proteins, we expect that some yet-uncharacterized NMD-causing proteins are part of our interaction network. We examined in more detail the candidate genes for the orphan LGMDs. In addition to the causative genes for 4 dominant and 17 recessive LGMD forms that are known so far, genetic linkage analyses have been used to map loci for three new LGMDs. These orphan LGMD loci contain between 13 and 45 genes, with a total of 2 genes encoding proteins of the LGMD-centered interactome. Consequently, FLNC and protein transport protein Sec31A (SEC31A) were isolated as unique candidates for LGMD1F and LGMD1G, respectively. No gene from the LGMD-centered interactome was found within the LGMD1E locus.

### Properties of the LGMD-centered network and biological functions of the proteins

To investigate which biological functions are associated with the LGMD-centered network, we performed a GO term enrichment analysis. In this analysis, we compared the LGMD dataset to a high-confidence (HC) network encompassing the most meaningful PPIs as defined with combined results from the experimental and computational analyses described above and a literature-based network.

The HC dataset was built by extracting from the LGMD-centered map the 491 PPIs classified in the PBS-A, -B, or -C categories plus PPIs from the PBS-D and -E categories for which we obtained additional evidence of the interaction. First, we added the 22 of our Y2H interactions from the PBS-D or -E categories that have been described in other studies (Additional file 1: Table S1). Second, PPIs from the PBS-D or -E categories were added to the HC dataset if experimental results from co-immunoprecipitation, immunofluorescence or PLA as described above confirmed the Y2H interaction. In total, 20 experimentally cross-validated interactions from the PBS-D category were included in the HC dataset. Third, were also included in the HC network, 107 pairs of interacting proteins from the PBS-D or -E categories that either share a BP GO annotation or share a CC and a MF GO annotation. Finally, since our dataset unraveled a high average level of relationship between NMD proteins, 65 PPIs in the PBS-D and -E categories and involving a NMD prey protein were added to the HC network. The resulting HC dataset consists of 497 proteins and 705 PPIs (Additional file 1: Table S1) and includes 174 and 40 PPIs from the PBS-D and -E categories, respectively.

The literature-based dataset was constructed by combining all direct protein-protein interactions reported in the literature for proteins of the HC dataset or LGMD-causing proteins that were not included in our initial set of baits. It is interesting to note that only two interactions for the five LGMD glycosyltransferases have been identified in previous reports [between Fukutin and protein O-linked-mannose beta-1,2-N-acetylglucosaminyltransferase 1 (POMGnT1) and between protein O-mannosyl-transferase 1 (POMT1) and 2 (POMT2)]. Since the glycosyltransferases are all described or predicted to be single or multipass membrane proteins, the rarity of PPI and their absence in our LGMD-centered network are possibly explained, since identification of interactions in the vicinity of membranes has proven to be difficult with the Y2H method. We searched for previously published experimental binary and direct interactions using the iRefWeb interface and identified 2675 direct binary interactions (Additional file 6: Table S5). This literature-based network consists of 2239 proteins and 3304 PPIs.

Characteristics of the three networks were analyzed (Table 4) [31]) and showed a connectivity of 0.022, 0.024 and 0.041 for the original and HC LGMD-centered networks and the literature-based network, with the proteins being associated with 19.7, 9.7 and 25.4 PPIs on average, respectively. Network heterogeneity, a parameter that reflects the tendency of a network to contain hub nodes was 2.895, 1.964 and 3.833 for the original and HC LGMD-centered networks and the literature-based network, respectively.

**Table 4:** Comparison of the three networks

	**Original**	**HC**	**Literature-based**
Number of proteins	1018	497	2239
Number of PPIs	1492	705	3304
Average number of partners per bait protein	19.7	9.7	25.4
Mean connectivity	0.024	0.022	0.041

*HC*: high-confidence; *PPI*: protein-protein interaction.

**Figure 7:**
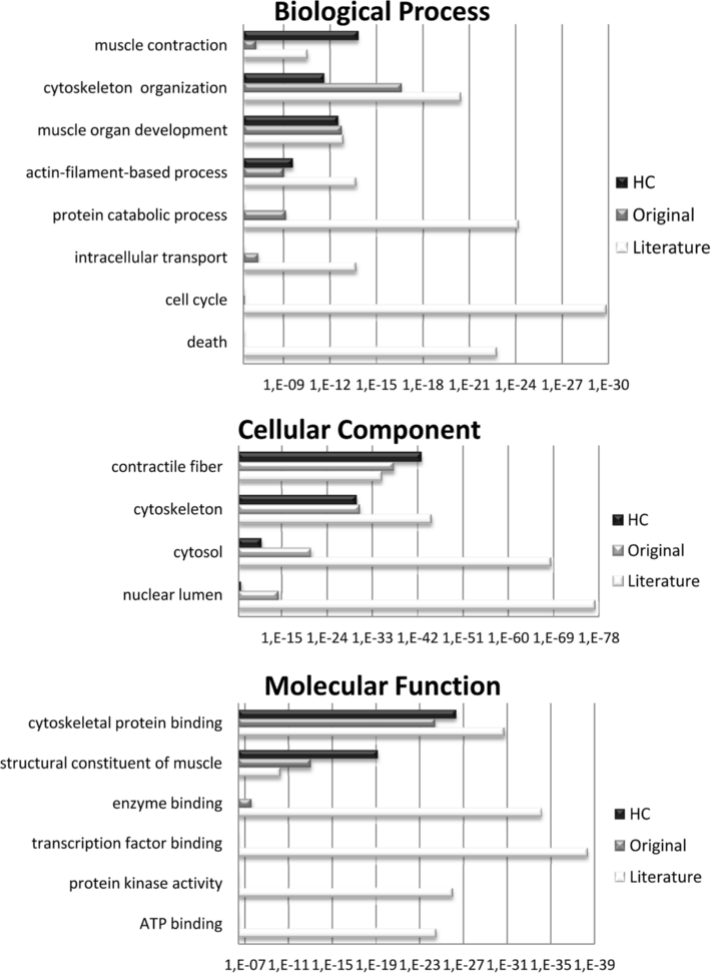
**GO enrichment analysis of the three datasets compared to the human proteome.** A GO term enrichment analysis of the three datasets (LGMD-centred, HC and literature-enriched datasets) was performed using the DAVID web-resource and an appropriate level of abstraction of the annotations, The bar charts depict enriched GO terms of the three datasets compared to the human proteome in the three branches of the GO structure as a function of the EASE score.

Using the DAVID web-resource and an appropriate level of abstraction of the annotations, we performed a GO term enrichment analysis of the three datasets in order to check if they presented any specificity in terms of biological process, cellular localization or molecular function of their proteins as compared to the human proteome (Figure 7). Not unexpectedly, they show an over-representation of proteins involved in muscle biology, even quite exclusively for both the original and HC LGMD-centered networks that share very similar profiles (Figure 7). The biological processes with most striking enrichments in all datasets are muscle contraction, cytoskeleton organization and muscle organ development. The cellular components with the highest representation include contractile fibers and cytoskeleton. The enriched molecular functions are cytoskeletal protein binding and structural constituents of muscle protein. In addition to these terms, the literature-based network shows additional terms with a significant enrichment of proteins involved in cell cycle and death, localized in the cytosol and the nucleus and with broader molecular functions (binding of enzyme, transcription factor or ATP and protein kinase).

A table explaining the component of all the supplements is added as Table 5.

**Table 5:** Additional file contents

**Additional file**	**Contents**
Additional file 1: Table S1	gives a comprehensive list of all the identified PPI
Additional file 2: Table S2	gives comprehensive information about the baits and Y2H screenings
Additional file 3: Table S3	gives the list of all the SIDs
Additional file 4: Table S4	presents the Human diseases associated with the proteins of the network
Additional file 5: Figure S1	presents a sub-network composed of the disease-related proteins only
Additional file 6: Table S5	gives the list of the PPIs for the literature-based network

*PPI*: protein-protein interaction; *SID*: selected interacting domain; *Y2H*: yeast-two hybrid.

## Discussion

Our Y2H experimental strategy was a large-scale protein domain-based approach using a methodology that has been previously successfully implemented [14]. As compared to approaches where baits are screened against full-length prey proteins, a domain-based approach offers several advantages including the possibility of narrowing down interaction domains and of reducing the false negative discovery rate by allowing a more efficient folding of domains [32]. A similar approach used in the field of ataxia identified interactions that were missed in a previous full-length protein-based approach [33]. Interestingly, this domain-based approach enabled us to analyze possible bias in interacting domains. We found that the three most frequent domains present in the SIDs (Immunoglobulin-like, Zinc Finger and Fibronectin domains) are also amongst the most frequent ones in the human proteome, emphasizing how important biomolecular interactions are for cellular processes. Two other domains (Ankyrin repeat and Nebulin motif) were frequently reported in the SIDs whereas they are rare in the human proteome. These domains were established as useful for meeting the demands of skeletal muscle physiology and constraints [34,35]. Auto-binding capacities of ankyrin repeats lead to mechanical-resistant dimers and nebulin-like domains play a role in the regulation of muscle contraction, especially through their interaction with actin and the thin filament. On the other hand, two domains frequently found in the human proteome (EGF-like and P-loop domains) are underrepresented in our set of interacting domains. This observation could be explained either by the function of the domain; the P-loop motif is known to be involved in hydrolysis of ATP and GTP but not in protein-protein interaction; or by a technical bias as in the case of the EGF-like domain which principally serves as an interacting domain within extracellular protein modules. The underrepresentation of this last domain is probably related to the fact that the LGMD proteins are intracellular proteins with the exception of sarcoglycans but for which the intracellular domain was selected for the Y2H screening.

The utility of any network obviously depends on the quality of the data. Our approach resides in the exploitation of random-primed cDNA libraries constructed from human skeletal muscle poly(A) RNA. It minimizes the risk of detecting interactions between proteins that would not be co-expressed in the muscle tissue and therefore should reduce the number of biological false positives. In addition, assignment of a PBS score using a statistical method allowed us to classify each PPI identified in our Y2H assays into five predictive categories, ranging from the most reliable interactions to possible technical false positives. The PBS score was previously demonstrated to successfully predict the reliability of a PPI on a subset of Y2H interactions where the authors experimentally confirmed 79% of all PPIs from the PBS-A, -B or -C categories by pull-down or co-immunoprecipitation assays [14]. The PBS takes into account both local parameters, such as the number of identical or independent fragments found for each partner and global information derived from the entire network such as reciprocal interactions, highly connected domains etc… Our screens identified a high percentage of the PBS-D interactions (53.6%) which reflects the high complexity of the constructed muscle library and the fact that this library is screened to saturation. As a consequence, the rate of false negatives should be extremely low, as even rare transcripts in the library, or weak or transient interactions, could be detected. In line with the notion that PBS-D interactions might be more difficult to detect, a higher proportion of interactions from the ABC categories than from the D category can be confirmed by other methods, such as co-immunoprecipitation or pull-down [14]. However, they could also represent false positive interactions and should be considered with caution. Finally, over 210 publications (full list available http://www.hybrigenics-services.com/publications/index/list). Of note, 27% of the publications reporting the functional validation of protein interactions identified using the same Y2H methodology correspond to the validation of a D interaction. This indicates that a significant proportion of such low confidence Y2H interactions are functionally relevant.

In the present study, we further confirmed the relevance of the PBS score with computational and experimental evidence. First, nearly a fifth of the PBS-A PPIs were found to have been previously reported whereas this figure falls to less than 5% when considering the whole interactome. Second, co-immunoprecipitation assays confirmed as positive 11 out of 16 technically conclusive tests (69%) and PLA validated 34 of the 44 tested interactions (77%). Although there is often limited overlap between studies [2,36], probably in part because of the spatial and temporal aspects of the proteome interaction, we compared our results obtained for DYSF with a recent study that analyzed the composition of DYSF complexes in cultured myoblasts, myotubes and skeletal muscle tissues by mass spectrometry and bioinformatics methods [37]. Interestingly, 19 (14%) of the 136 DYSF interactions identified by our large-scale Y2H screening were also found in the de Moree’s study, a ratio that is a little higher than what is usually observed [3]. In contrast, only 3 interactions (TTN, ACTN2 and DES) were found in a study based on Fisher’ method [38] and none in another *in silico* study [39].

Remarkably, results of our Y2H screens led to a single connected network where the different LGMD proteins are highly connected. The strong inter-connectivity between LGMD proteins is illustrated by a high number of direct interactions. This was quite surprising since, even if the different LGMD forms share seemingly close clinical phenotypes, the LGMD-causing proteins have been described to have quite diverse locations and biological functions. In addition to identifying a remarkable number of direct interactions between LGMD proteins, mining the data revealed that LGMD proteins belong to a highly connected network of interacting proteins with, in particular, the sarcomeric proteins ACTN2, MYBPC1, MYOM1 and MYOM2 identified as hub proteins sharing the highest number of links with the LGMD proteins. Interestingly, these proteins are structural proteins located at key places on the sarcomere, the Z-disc, the N2A-line and the M-band. GO analyses further support the crucial place of the cytoskeleton in the connections between LGMD proteins. Taken together, these data suggest that common molecular mechanisms underlie the pathogenesis of these diseases and highlight the sarcomere as an important platform for skeletal muscle homeostasis and myofiber survival.

We expect that our interaction map can serve as a new tool to accelerate discovery of the causative mutated genes for orphan LGMDs or for other orphan NMDs already or not yet described as well as to identify modifier genes. Examination of the chromosomal location of all the genes coding for the proteins that are part of our original interactome map revealed putative candidates for orphan NMDs of which *FLN* and, *SEC31A* appeared to be of particular interest for LGMD1F and LGMD1G, respectively. Defects in the *FLNC* gene coding for an actin-binding protein, are already known to cause myofibrillar myopathies [40] but the gene was supposedly excluded as being involved in the pathogenesis of LGMD1F [41]. Nevertheless, non-coding sequences were not fully investigated for pathogenic mutations and, as mentioned by the authors, the gene remains a possible candidate. The *SEC31A* gene coding for a component of a protein complex responsible for vesicle budding from the endoplasmic reticulum [42], has not been associated with any disease. It is an interesting candidate since LGMD1G is associated with progressive limitation of fingers and toes flexion [43] and SEC31A has been linked to collagen secretion [44]. In addition to the LGMD proteins, there is a high proportion of proteins involved in congenital and metabolic myopathies in our network, therefore it is very likely that causative genes for genetically uncharacterized forms of these two groups of diseases lie within our interactome.

An interesting outcome of our study is to provide new PPIs that further support and extend previous findings and pinpoint new pathways of interest that could be affected in LGMD. For example, two novel interactions of calpain 3 (CAPN3) with, ring finger protein 167 (RNF167), an E3 ubiquitin-protein ligase, and the proteasome maturation factor (POMP) are of particular interest considering a previous report indicating that CAPN3 acts upstream of the ubiquitin-proteasome system [45]. For DYSF, a number of new interacting partners can be categorized in three different cellular processes: endocytosis, microtubule-related transport and regulation of gene expression. The first two pathways fit well with previous knowledge about DYSF functions but, interestingly, the third pathway indicates a new possible role for this protein. Another interesting finding in view of the fact that the pathogenesis of SGCG deficiency does not seem strictly related to membrane stability [46,47], is the possible relationship of this protein with energy controlling pathways since interaction with proteins involved in glycolysis or glycogenolysis (enolases 1 and 3 and PYGM) or in the TCA cycle (SUCLG2, ACO1) was identified. Finally, several partners detected for TCAP suggest that it may play a role in the maintenance of genome integrity, in accordance to the recent report showing a relationship between TCAP and p53 turn-over [48]. These elements provide new avenues to explore for a better understanding of the pathophysiology of the various forms of LGMD.

In conclusion, this study presents new interacting partners for LGMD proteins and other proteins known to be involved in NMD. In this sense, it has the potential to reveal new candidate genes for NMD but also modifiers of the phenotype. This broad dataset should also help to take a step further towards the understanding of skeletal muscle tissue. In particular, it will help to improve our knowledge about the cellular functions and roles of NMD proteins in the muscle cell and about their participation in the diseases they trigger thereby speeding up the identification of putative drug targets.

## Abbreviations

Y2H: Yeast-two hybrid; PPI: Protein-protein interaction; HC: High-confidence; SID: Selected Interacting Domains; PBS: Predicted Biological Score; PLA: Proximity ligation assay; GO: Gene Ontology; BP: Biological processes; CC: Cell components; M F: Molecular functions; NMDs: Neuromuscular Disorders; LGMD: Limb-girdle muscular dystrophy; LGMD2: Limb-girdle muscular dystrophy, type 2; MDC1B: Congenital muscular dystrophy 1B; MPRM1: Autosomal dominant myopathy with proximal muscle weakness and early respiratory muscle involvement; HBM: Hyaline body myopathy; MPD3: Adult onset distal myopathy; MFM/ ARVC: Myofibrillar myopathy with arrythmogenic right ventricular cardiomyopathy; NEM6: Nemaline myopathy; MDRV: Distal myopathy with pes cavus and areflexia; MEAX: Myopathy with excessive autophagia.

## Competing interest

The authors report no competing interest.

## Authors’ contributions

GB designed and performed the experiments, analyzed data and wrote the paper. SM performed the experiments and analyzed data. KC, ND and MB designed the experiments and analyzed data. ND, EG, JBB, AB and LB performed the experiments. DS developed analytical tools. IR designed experiments, analyzed data and wrote the paper. All authors read and approved the final manuscript.

## Supplementary Material

Additional file 1: Table S1 Comprehensive list of PPI of the LGMD-centered network. Column A: Bait gene symbol; Column B: GI number of reference for bait sequence (the name of the protein is indicated in Table S1) Additional file 2: Table S2; Columns C-D: HUGO Gene Nomenclature Committee (HGNC) and Uniprot identifiers for the bait (mapped by DAVID); Column E: Prey gene symbol; Column F: GI number of reference for prey sequence; Column G: Genbank notes for prey protein; Columns H-I: HGNC and Uniprot identifiers for the prey (mapped by DAVID); Column J: PBS category (A to E) for the Y2H PPI; Column K: PPI belonging to the HC network (Y=yes); Column L: Reciprocal hits in our experiment (Y=yes); Column M: Pubmed ID for previously described PPI in mammals; Column N: PPI experimentally validated by immunoprecipitation (IP_pos), co-localisation assays (Coloc_pos) or proximity ligation assays (Duolink_pos). The negative results are also indicated (_neg), Columns O-Q: GO annotation for pairs of proteins sharing a BP, CC and MF cluster, respectively; Column R: Shared NMD associated with the prey protein; Column S: OMIM of the associated NMD. Click here for file

Additional file 2: Table S2 Comprehensive bait and Y2H screenings information. a) Interaction was reciprocally found (A<->B) (b) TTN novex-3 variant was found as prey. Click here for file

Additional file 3: Table S3 List of SIDs. Column A: PBS category; Column B: Bait gene symbol; Column C: Bait GI number; Columns D-E: HGNC and Uniprot identifiers for the bait (mapped by DAVID; Column F: Bait coordinates (aa) on the gi sequence translated product; Column G: Prey gene symbol; Column H: Prey GI number; Columns I-J: HGNC and Uniprot identifiers for the prey (mapped by DAVID); Columns K-L: SID coordinate on the prey gi sequence translated product (first and last aa, respectively); Column M-O: For PPIs from the PBS-A, -B and -C categories, Interpro domains including the SID, included within the SID or overlapping with the SID, respectively. Click here for file

Additional file 4: Table S4 Human diseases associated with the proteins of the network. Column A: Gene symbol; Column B: GI number of the bait or prey sequence; Column C: Human genetic conditions associated with the protein; Column D: OMIM number (ND= not defined); Column E: Classification as NMD (Y=yes, N=no); Column F: Classification of NMD according to the Neuromuscular Disorders Online Gene Table (http://www.musclegenetable.fr) 1 Muscular dystrophies, 2 Congenital muscular dystrophies, 3 Congenital myopathies, 4 Distal myopathies, 5 Other myopathies, 6 Myotonic syndromes, 7 Ion channel muscle diseases, 8 Malignant hyperthermia, 9 Metabolic myopathies, 10 Hereditary cardiomyopathies, 11 Congenital myasthenic syndromes, 12 Spinal muscular atrophies, 13 Hereditary ataxia, 14 Hereditary motor and sensory neuropathies, 15 Hereditary paraplegia, 16 Other NMD. Click here for file

Additional file 5: Figure S1 Diseases related-protein network. NMD-related proteins are depicted as blue node ovals and non-NMD-related proteins are depicted as yellow node ovals. Interactions between pairs of disease-related proteins are depicted by edges with colors according to the PBS category (PBS-A: red, PBS-B: dark blue, PBS-C: green, PBS-D: light blue, PBS-E: light pink). Only proteins that show interactions with diseases-related proteins are presented. Click here for file

Additional file 6: Table S5 List of PPIs for the literature-based network. Column A: Gene symbol for the protein of the interactome; Columns B-C: Gene symbol and HGNC identifier for the interacting partner from the Intact database or the interactome; Column D: Name of the interacting partner. Click here for file
