# Supplementary figures and images for: A human skeletal muscle interactome centered on proteins involved in muscular dystrophies: LGMD interactome
Source: Skelet Muscle. 2013 Feb 15;3:3. doi: 10.1186/2044-5040-3-3 (PMC3610214; doi:10.1186/2044-5040-3-3)

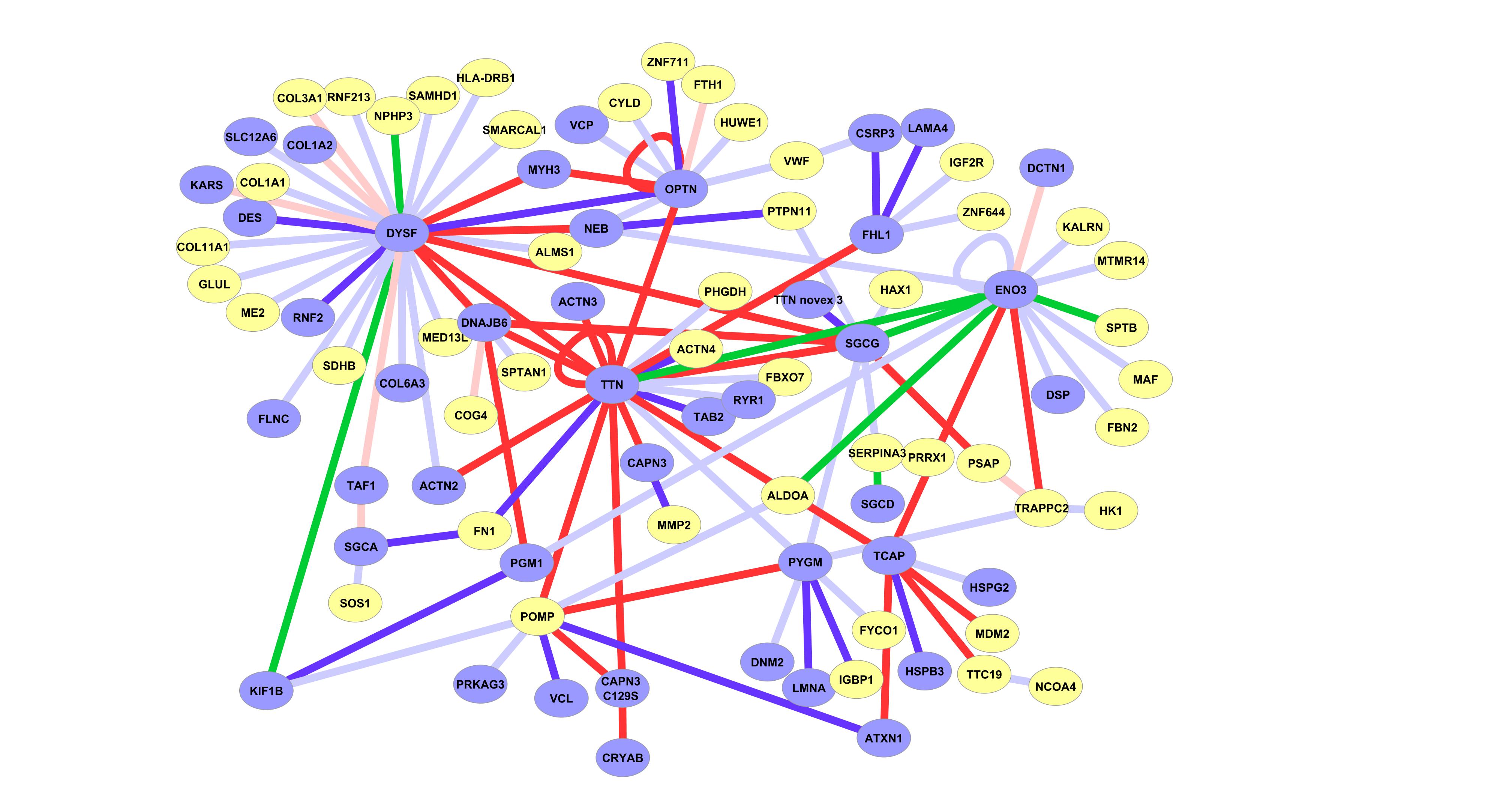

Supplement: Additional file 5: Figure S1 — Diseases related-protein network. NMD-related proteins are depicted as blue node ovals and non-NMD-related proteins are depicted as yellow node ovals. Interactions between pairs of disease-related proteins are depicted by edges with colors according to the PBS category (PBS-A: red, PBS-B: dark blue, PBS-C: green, PBS-D: light blue, PBS-E: light pink). Only proteins that show interactions with diseases-related proteins are presented. [file 2044-5040-3-3-S5.jpeg]
